# Large Language Models in Cosmetic Dermatology

**DOI:** 10.1111/jocd.70044

**Published:** 2025-02-12

**Authors:** Marina Landau, George Kroumpouzos, Mohamad Goldust

**Affiliations:** ^1^ Arena Dermatology and Department of Plastic Surgery Shamir Medical Center Be'er Ya'akov Israel; ^2^ Department of Dermatology Warren Alpert Medical School at Brown University Providence Rhode Island USA; ^3^ GK Dermatology PC South Weymouth Massachusetts USA; ^4^ Department of Dermatology Yale University School of Medicine New Haven Connecticut USA

**Keywords:** aesthetic dermatology, artificial intelligence, cosmetic dermatology, large language model

Dear Editor,

Artificial intelligence (AI), particularly large language models (LLMs) like ChatGPT and Gemini, is expanding healthcare, specifically in the field of cosmetic dermatology. These advanced AI systems are designed to process and generate human‐like text by analyzing vast amounts of data. Through natural language processing (NLP), LLMs offer innovative solutions that improve patient care, facilitate clinical workflows, and accelerate research efforts. However, while these models hold significant potential, it is crucial to address their limitations, as well as the ethical considerations and regulatory challenges associated with their use, to ensure responsible implementation [1, 2].

At their core, LLMs are sophisticated computer programs capable of understanding and generating human‐like text. Imagine conversing with a knowledgeable assistant who can quickly summarize medical research, suggest treatment options, or answer complex questions. LLMs achieve this by learning patterns from extensive datasets, including books, articles, and scientific journals. With this training, they can synthesize information and provide coherent responses to user inquiries [3].

LLMs are expanding several aspects of cosmetic dermatology. One key application lies in patient education. By simplifying complex medical terminology, LLMs make cosmetic procedures, such as dermal fillers, neurotoxins, and laser therapies, more accessible to patients. This improved communication enables patients to make informed decisions about their care. Additionally, these tools have the potential to bridge knowledge gaps in underserved communities, although challenges like limited digital proficiency and internet access still persist.

Another critical application is personalized treatment planning. LLMs can analyze patient histories, procedural risks, and desired outcomes to provide evidence‐based recommendations. For instance, they may suggest combining microneedling with platelet‐rich plasma therapy to achieve optimal skin rejuvenation. While this personalization can enhance treatment outcomes, it requires clinician oversight to ensure that recommendations match individual patient needs and are free from bias.

LLMs also contribute to administrative efficiency in dermatology practices. Integrating these tools into electronic medical record (EMR) systems can automate tasks such as clinical note‐taking, scheduling, and insurance coding. This automation reduces administrative burdens, allowing clinicians to devote more time to direct patient care [4].

Follow‐up care and monitoring are additional areas where LLMs show efficiency. These models can interact with patients' postprocedure, ensuring adherence to recovery protocols, identifying potential complications, and assessing satisfaction levels. These capabilities enhance continuity of care and provide valuable feedback for clinicians to improve their services.

In training and simulation, LLMs are advancing dermatology education. Virtual platforms powered by AI simulate patient scenarios and provide real‐time feedback, enabling trainees to refine diagnostic and procedural skills in a safe and interactive environment. This application is particularly beneficial for mastering emerging techniques in cosmetic dermatology.

Emerging technologies, such as predictive modeling and augmented reality (AR), are also benefiting from the integration of LLMs. For example, tools like Gemini can combine text and image analysis to simulate procedural outcomes, such as the effects of laser resurfacing. This capability enhances communication between clinicians and patients, setting realistic expectations and improving satisfaction.

Despite their potential, large language models (LLMs) present several challenges. One major concern is the risk of inaccuracies and biases. Training datasets that lack diversity can lead to inequitable or unreliable recommendations. For example, some AI tools have been misrepresented as using LLM technology, such as Skinive and AestheticPro AI, which could mislead users about their actual capabilities.

Ethical concerns also arise regarding patient data privacy. Sharing sensitive medical information with AI systems necessitates robust encryption and compliance with regulations such as HIPAA and GDPR. Without strict protections, these systems could unintentionally compromise patient confidentiality.

Accessibility is another critical issue. Underserved populations, who could benefit significantly from AI advancements, often face barriers such as limited internet access, low digital proficiency, and high costs. Developing cost‐effective and offline‐compatible tools is essential to address these disparities and ensure equitable access.

High subscription costs associated with advanced AI platforms may prevent smaller practices or underfunded institutions from adopting these technologies. Addressing this financial barrier is crucial to preventing healthcare inequities.

Human oversight remains vital in the use of LLMs. These tools should complement, not replace, clinical expertise. Ensuring that AI outputs are validated by clinicians helps maintain accuracy and provides trust with patients.

To ensure the safe and effective use of LLMs, clinical validation is essential. Tools like DermaGPT and DeepSkinAI must undergo rigorous testing to evaluate their reliability in real‐world settings. Additionally, customizing these technologies with established regulatory frameworks, such as FDA guidelines, can standardize approval processes and decrease potential risks.

Collaboration among AI developers, dermatologists, and policymakers is necessary to address these challenges. This partnership can help develop ethical guidelines, enhance data quality, and promote equitable access to these transformative tools [5].

The responsible use of LLMs requires a multi‐directional approach. First, ensuring data quality by providing diverse and representative datasets can minimize biases. Second, conducting peer‐reviewed studies to validate AI tools enhances their credibility. Third, investing in affordable solutions can help bridge accessibility gaps, particularly in underserved areas. Finally, educating both clinicians and patients about the capabilities and limitations of LLMs is crucial for providing informed usage and trust.

Large language models such as ChatGPT and Gemini are set to transform cosmetic dermatology by improving patient engagement, optimizing clinical workflows, and driving innovation in research. However, realizing their full potential requires addressing their limitations, including biases, ethical concerns, and accessibility challenges. Through collaboration and innovation, these technologies can deliver on their promise while upholding equity, trust, and clinical integrity.

## Author Contributions

We confirm that the manuscript has been read and approved by all the authors, that the requirements for authorship as stated earlier in this document have been met and that each author believes that the manuscript represents honest work.

## Consent

The authors have nothing to report.

## Conflicts of Interest

The authors declare no conflicts of interest.

## Data Availability

Data sharing not applicable to this article as no datasets were generated or analysed during the current study.
